# Supplementary material for: Microbiological and Immunological Markers in Milk and Infant Feces for Common Gastrointestinal Disorders: A Pilot Study
Source: Nutrients. 2020 Feb 27;12(3):634. doi: 10.3390/nu12030634 (PMC7146151; doi:10.3390/nu12030634)
Supplement: Supplementary file 1 [file nutrients-12-00634-s001.pdf]

Microbiological and Immunological Markers in Milk and Infant Feces for Common Gastrointestinal Disorders: A Pilot Study

Marina Aparicio [1], Claudio Alba [2], Proctocolitis Study Group of CAM Public Health Area 6 [3], Juan Miguel Rodríguez [1] and Leonides Fernández [2,*]

Supplementary Table 1. Global comparison of the frequencies of detection of immunological compounds in milk (*n* = 25) and fecal (*n* = 28) samples.

Immunological compound	Feces (n/N)	Milk (n/N)	*p**
**Innate immunity**			
IL1β	24/28	18/25	0.313
IL6	**27/28**	15/25	0.002
IL12	**28/28**	14/25	<0.001
IFNγ	3/28	0/25	0.238[a]
TNFα	18/28	10/25	0.102
**Acquired immunity**			
IL2	**28/28**	2/25	<0.001[a]
IL4	1/28	**9/25**	0.004[a]
IL10	**21/28**	10/25	0.013
IL13	10/28	**17/25**	0.028
IL17	**28/28**	3/25	<0.001
**Chemokines**			
IL8	16/28	**24/25**	0.001
MCP1	6/28	**20/25**	<0.001
MIP1β	17/28	**25/25**	<0.001
**Hematopoyetic factors**			
IL5	**28/28**	1/25	<0.001[a]
IL7	12/28	6/25	0.245
GCSF	**25/28**	8/25	<0.001
GMCSF	**20/28**	10/25	0.028

GCSF, granulocyte colony-stimulating factor; GMCSF, granulocyte–macrophage colony-stimulating factor; IFNγ, interferon-γ; IL, interleukin; MCP1, macrophage–monocyte chemoattractant protein-1; MIP1β, macrophage inflammatory protein-1β; TNFα, tumor necrosis factor-α. n/N, number of samples in which the immunological compound was detected/total number of samples assayed. * Chi-squared tests (or Fisher's exact-tests, marked with a) were used to evaluate differences in detection frequencies of the analyzed parameters.

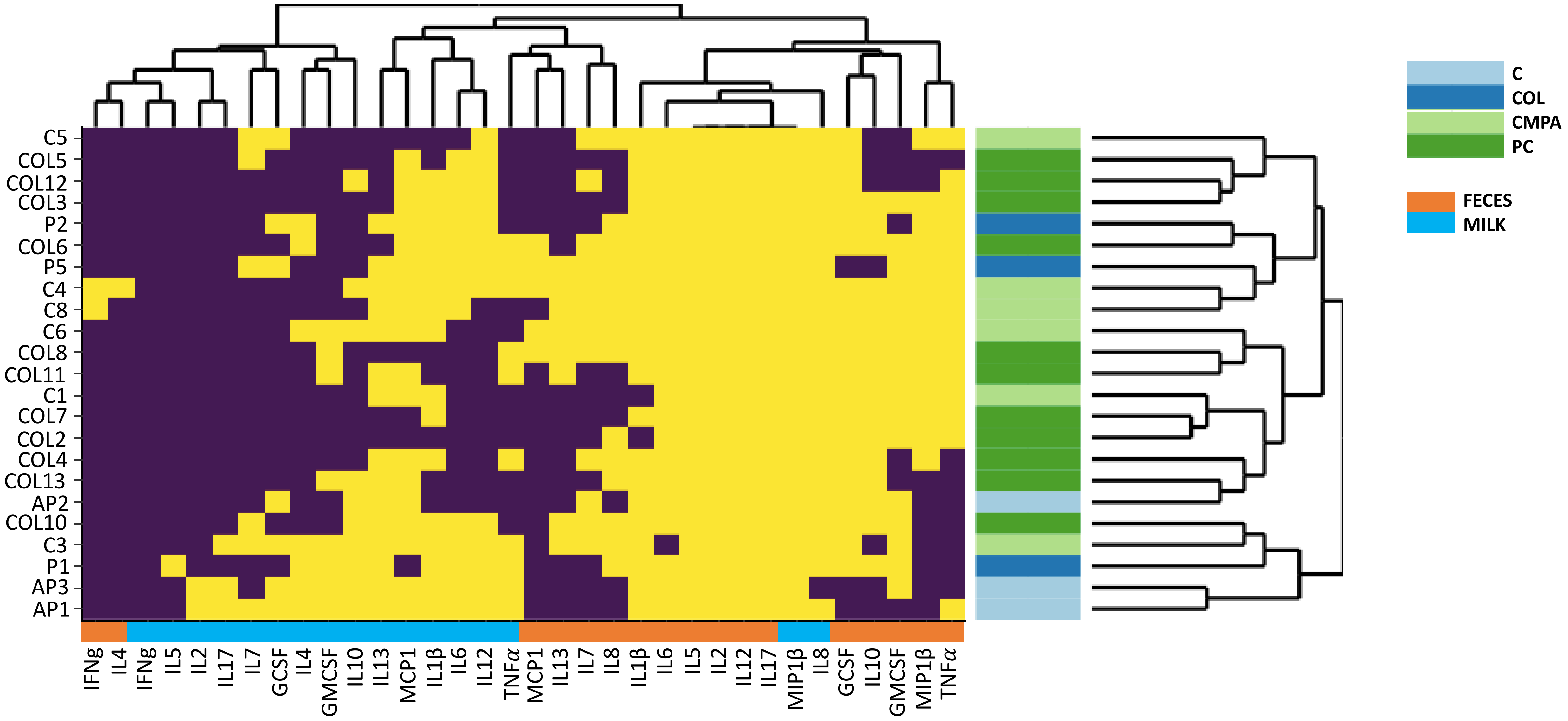

Supplementary Figure 1. Cluster heatmap of the immunological factors present in the fecal and milk samples (*n* = 34) from the different participants included in the study. Blue color indicates the presence of a factor, and yellow color indicates its absence in the sample. The color to the right indicates the study group: control (C, light blue), colic (COL, deep blue), cow's milk protein allergy (CMPA, light green), and proctocolitis (PC, deep green). The immunological factor and type of sample is indicated at the bottom: feces (orange), and milk (cyanide). Heatmap and dendrogram indicate the absence/presence of the different immunological compounds showing different immunological profiles (variables clustered on the vertical axis). Patients were clustered using dist_method=NULL and hclust_method=NULL. GCSF, granulocyte colony-stimulating factor; GMCSF, granulocyte–macrophage colony-stimulating factor; IFNg, interferon-γ; IL, interleukin; MCP1, macrophage–monocyte chemoattractant protein-1; MIP1β, macrophage inflammatory protein-1β; TNFα, tumor necrosis factor-α.
